# PACAP regulates VPAC1 expression, inflammatory processes and lipid homeostasis in M1- and M2-macrophages

**DOI:** 10.3389/fcvm.2023.1264901

**Published:** 2023-10-13

**Authors:** Roman Witzel, Annika Block, Solvey Pollmann, Leandra Oetzel, Fenja Fleck, Gabriel A. Bonaterra, Ralf Kinscherf, Anja Schwarz

**Affiliations:** Department of Medical Cell Biology, Institute of Anatomy and Cell Biology, Philipps-University of Marburg, Marburg, Germany

**Keywords:** PACAP, VPAC1, oxLDL, macrophages, atherosclerosis, inflammation

## Abstract

**Background:**

Pituitary adenylate cyclase-activating polypeptide (PACAP) acts as an anti-atherogenic neuropeptide and plays an important role in cytoprotective, as well as inflammatory processes, and cardiovascular regulation. Therefore, the aim of this study is to investigate the regulatory effects of PACAP and its receptor VPAC1 in relation to inflammatory processes and lipid homeostasis in different macrophage (MΦ) subtypes.

**Methods:**

To investigate the role of PACAP deficiency in the pathogenesis of atherosclerosis under standard chow (SC) or cholesterol-enriched diet (CED) *in vivo*, PACAP^−/−^ mice were crossbred with ApoE^−/−^ to generate PACAP^−/−^/ApoE^−/−^ mice. Lumen stenosis in the aortic arch and different MΦ-subtypes were analyzed in atherosclerotic plaques by quantitative immunohistochemistry. Undifferentiated bone marrow-derived cells (BMDC) from 30-weeks-old ApoE^−/−^ and PACAP^−/−^/ApoE^−/−^ mice were isolated, differentiated into BMDM1- and BMDM2-MΦ, and incubated with oxidized low-density lipoprotein (oxLDL). In addition, PMA-differentiated human THP-1 MΦ were further differentiated into M1-/M2-MΦ and subsequently treated with PACAP38, the VPAC1 agonist [(Ala11,22,28)VIP], the antagonist (PG 97–269), and/or oxLDL. Uptake/accumulation of oxLDL was analyzed by oxLDL-DyLight™488 and Bodipy™ 493/503. The mRNA expression was analyzed by qRT-PCR, protein levels by Western blot, and cytokine release by ELISA.

**Results:**

*In vivo*, after 30 weeks of SC, PACAP^−/−^/ApoE^−/−^ mice showed increased lumen stenosis compared with ApoE^−/−^ mice. In atherosclerotic plaques of PACAP^−/−^/ApoE^−/−^ mice under CED, immunoreactive areas of VPAC1, CD86, and CD163 were increased compared with ApoE^−/−^ mice. *In vitro*, VPAC1 protein levels were increased in PACAP^−/−^/ApoE^−/−^ BMDM compared with ApoE^−/−^ BMDM, resulting in increased TNF-α mRNA expression in BMDM1-MΦ and decreased TNF-α release in BMDM2-MΦ. Concerning lipid homeostasis, PACAP deficiency decreased the area of lipid droplets in BMDM1-/M2-MΦ with concomitant increasing adipose differentiation-related protein level. In THP-1 M1-/M2-MΦ, the VPAC1 antagonist increased the uptake of oxLDL, whereas the VPAC1 agonist decreased the oxLDL-induced intracellular triglyceride content.

**Conclusion:**

Our data suggest that PACAP via VPAC1 signaling plays an important regulatory role in inflammatory processes in atherosclerotic plaques and in lipid homeostasis in different MΦ-subtypes, thereby affecting foam cell formation. Therefore, VPAC1 agonists or PACAP may represent a new class of anti-atherogenic therapeutics.

## 1. Introduction

Atherosclerosis is a chronic inflammatory disease (1) enhanced not only by lipid deposits in the arterial wall, but also by cellular waste products, calcium or fibrin. However, these factors play only a minor role in our study. At arterial branch points and bends, circulating lipoprotein particles can enter the arterial wall and accumulate in the intima, promoting the development of atherosclerotic lesions.

Low-density lipoprotein (LDL), especially in its modified forms, is the major contributor to lipid accumulation in atherosclerotic lesions (2). In this context, modifications of LDL, such as oxidation, trigger an immune response leading to the formation of circulating LDL-containing immune complexes that are exceedingly atherogenic (3, 4). Moreover, the progression of atherosclerosis is associated with phenotypic diversity of MΦ. In general, MΦ are a fundamental immune system component and can be classified into two subtypes. The pro-inflammatory or immunosuppressive M1-MΦ are classically activated and produce inflammatory cytokines such as tumor necrosis factor-alpha (TNF-α) (5–8). M1-MΦ are a predominant phenotype in rupture-prone zones, plaque progression and advanced lesions, whereas they are abundant in unstable plaques (9, 10). The anti-inflammatory M2-MΦ are important for tissue and wound healing (6, 8, 11) and present in the early stages of atherosclerotic plaques (12–14). Thus, identifying the phenotype is also used as a marker of plaque stability (15). MΦ in atherosclerotic lesions are actively involved in the uptake and accumulation of lipoproteins, forming foam cells. The accumulation of foam cells contributes to further and increased lipid storage and, thus, to the progression of atherosclerotic plaque (16). Therefore, it is essential to understand and decipher the biomolecular and phenotypic diversity of MΦ to reveal their functions and roles in atherosclerotic plaques to develop specific therapies for atherosclerosis. In addition, the microenvironment of the atherosclerotic plaque (lipids, growth factors, cytokines, chemokines) regulates the MΦ subtypes in the plaque itself (31). By targeting the MΦ microenvironment of the atherosclerotic plaque, previous research shows potential strategies for selective treatment of atherosclerosis (3).

In 1989, the pituitary adenylate cyclase-activating polypeptide (PACAP) was isolated from ovine hypothalamic extracts and described in two amidated forms: PACAP27 and PACAP38 (17). PACAP is widely distributed in the peripheral and central nervous systems (18, 19). The vasoactive intestinal polypeptide (VIP), which is very similar to PACAP, and also PACAP regulate numerous biological activities by binding to specific plasma membrane receptors. These plasma membrane receptors belong to subfamily II within the superfamily of G protein-coupled receptors: (1) the type 1 and type 2 VIP receptors (VPAC1 and VPAC2), which bind VIP and PACAP with equal affinity (Kd = 1.0 nM), and (2) the PACAP receptor (PAC1), which is selective for PACAP (Kd = 0.5 nM) (20–23). The existence of VPAC1 receptor has been previously described in human monocytes (24), mouse peritoneal MΦ (25–27), and rat alveolar MΦ (28). Our previously published data show that PACAP deficiency in apolipoprotein (Apo)E knockout mice (ApoE^−/−^) mice (30 weeks under standard chow) promotes the development and progression of atherosclerotic plaques through the proatherogenic increase of inflammatory factors, autophagy, apoptosis, necroptosis, and fibrosis (29).

In this context, this study investigated the expression of PACAP and the PACAP receptor VPAC1 concerning lipid homeostasis and inflammatory processes in M1-/M2-MΦ. Our data show that PACAP regulates VPAC1 protein levels, which has implications for cellular inflammatory processes and affects lipid homeostasis in MΦ subtypes. Therefore, we consider it fundamental to further investigate the efficacy of VPAC1 or PACAP in regards to anti-atherogenic therapeutics.

## 2. Materials and methods

### 2.1. Animals

To generate PACAP knockout mice (PACAP^−/−^), PACAP was deleted from the PACAP gene locus in C57BL/6 mice (30). Then, PACAP knockout mice (PACAP^−/−^) were crossbred with ApoE^−/−^ (Charles River, Sulzfeld, Germany) to generate PACAP^−/−^/ApoE^−/−^ mice (29). For this study, only male homozygous PACAP^−/−^/ApoE^−/−^ and ApoE^−/−^ mice were used, which were fed either standard chow for 30 weeks (SC, LASQCdiet® Rod16 Rad; LASvendi, Soest, Germany) or were fed a cholesterol-enriched diet [CED; “western-type diet” (21% fat, 0.15% cholesterol, and 19.5% casein), Altromin GmbH, Lage, Germany] for an additional 20 weeks at 10 weeks of age. All animals had *ad libitum* access to water and feed in their cages which had a minimum area of 100 cm^2^ and an adequate enrichment device. The procedures were approved by the Regierungspräsidium Gießen (V54–19 c 2015 h 01 MR 20/26 No. 21/2014) and complied with the regulations for animal experiments at the Philipps-University Marburg.

### 2.2. Dissection and tissue harvesting

Mice 30 weeks of age were intraperitoneally analgized for tissue removal with a combination of ketamine (150 mg/kg) and xylazine (20 mg/kg) (29) and then weighed and measured. Local intercostal anesthesia was performed with lidocaine 2%. After opening the thorax, the tip of the left ventricle was opened and a cannula (8 G, B. Braun Melsungen AG, Melsungen, Germany) was inserted. The vasculature was perfused with a solution of phosphate-buffered saline (PBS) with 5 Ul/ml heparin (Liquemin® 25,000 Ul/5 ml, Roche, Grenzach, Germany) using an automatic syringe pump (Secura, B. Braun, Melsungen AG) at 30 ml (rate of 100 ml/h). The aortic arch was harvested using a binocular loupe, embedded in Tissue-Tek® (Sakura Finetek, Stauffen, Germany), and frozen in liquid nitrogen-cooled isopentane.

### 2.3. Genotyping

Genomic DNA was isolated from the mouse ear using a commercial kit (DNA Extraction Solution; PeqLab, VWR Company, Erlangen, Germany) according to the manufacturer's instructions (DirectPCR® lysis reagent ear; Peqlab, VWR International; Darmstadt, Germany). Homozygous transgenic mice were subsequently detected by polymerase chain reaction (PCR) using intron-spanning oligonucleotides (see Table 1) (29, 30).

**Table 1:** Oligonucleotides used for genotyping.

Primer	Sequence	Amplification length (bp)	Company
PACAP^−/−^			
PACAP-neoOF	5′-CAC CGG CCT TTA GGG ACC CTT GTA-3′	520 (+/+)	Eurofins Genomics, Ebersberg, Germany
PACAP-2R	5′-GCT ATT CGG CGT CCT TTG TTT TTA ACC C-3′	520bp + 320 bp (+/−)	
PACAP-PNT1R	5′-TAG GGG AGG AGT AGA AGG TGG CGC-3′	310 bp (−/−)	
ApoE^−/−^			
oIMR180-F	5′-GCC TAG CCG AGG GAG AGC CG-3′	245 bp	Eurofins Genomics, Ebersberg, Germany
oIMR182-R	5′-GCC GCC CCG ACT GCA TCT-3′	245 bp	

### 2.4. Morphometry and immunohistology

Cryosection series (6 µm) of the aortic arch were prepared for morphometric and immunohistological studies. The extent of atherosclerotic plaques in the aortic arch was measured by computerized morphometry. These images were analyzed and quantified using Fiji software (31). For this purpose, standard hematoxylin-eosin (HE) staining was performed. Further immunohistochemical staining was performed with the antibodies listed in Table 2. The lumen stenosis was determined by recording the lumen and plaque areas along the internal elastic lamina (or luminal plaque circumference) and calculating [[plaque area (µm^2^)]/[lumen area (µm^2^)] × 100% = lumen stenosis (%)] (29). Media thickness was determined by calculating the area of the lumen along the internal elastic lamina and the area along the outer elastic lamina by calculating [lumen area to outer elastic lamina (µm^2^)]—[lumen area to internal elastic lamina (µm^2^)] = area of media (µm^2^)]. Quantification of immunoreactive plaque area was assessed as described previously (29, 32).

**Table 2:** List of used antibodies.

Name	Catalog Number	Company	Dilution
Primary antibody			
Rabbit anti-mouse CD86	Orb10351	Biorbyt, Cambridge, UK	1:50
Rabbit anti-mouse CD163	Orb182468	Biorbyt, Cambridge, UK	1:100
Rabbit anti-mouse VIPR1 (VPAC1)	PA3-113	ThermoFisher scientific, Rockford, USA	1:500 (IHC); 1:1,000 (WB)
Rat anti-mouse CD68	MCA1957	AbD Serotec, Düsseldorf, Germany	1:100
Rabbit anti-ADFP	ab108323	Abcam plc., Cambridge, UK	1:1,000
Rabbit Anti-Tubulin	ab4074	Abcam plc., Cambridge, UK	1:6,000
Secondary antibody			
Goat anti-rabbit IgG HRP	ZRH1158	Linaris, Dossenheim, Germany	1:200
Goat anti-rat IgG HRP	STAR72	AbD Serotec, Düsseldorf, Germany	1:200
Donkey anti-rabbit IgG, HRP-linked F(ab’)2-fragment	NA934	GE Healthcare Life Science Freiburg, Germany	1:3,000

WB, western blot; IHC, immunohistochemistry.

### 2.5. Cell culture

#### 2.5.1. MΦ from human leukemic monocyte cell line THP-1

The human leukemic monocyte cell line THP-1 (Leibniz Institute DSMZ, Braunschweig, Germany) was used. The THP-1 cells were frequently used as a model of monocyte/MΦ cell lineage (33) and routinely used in atherosclerosis research (29, 34). THP-1 cells were cultured in RPMI-1640 medium (Capricorn Scientific GmbH, Ebsdorfergrund, Germany) supplemented with penicillin and streptomycin (Capricorn Scientific GmbH) as well as 10% fetal bovine serum (Capricorn Scientific GmbH). Cells were cultured at 37°C in a 5% CO_2_ environment, with a medium change every 2–3 days. All experiments were performed using cells at the 9th passage or lower. Monocyte differentiation in M0-MΦ with 100 nM phorbol-12-myristate-13-acetate [PMA, (Sigma-Aldrich Chemie GmbH Munich, Germany)] was performed as described by Ackermann et al. (34).

#### 2.5.2. MΦ from bone marrow-derived cells

At the age of 30 weeks, mice were weighed, and anesthetized by inhalation of isoflurane. The body size was measured by measuring the length from nose-tip to tail-base before euthanasia by neck dislocation. The procedures were in accordance with the animal experiment regulations of the Philipps-University Marburg (Ex 17/2022). Bone marrow-derived cells (BMDC) were isolated postmortem from femurs of mice as described by Amend et al. (35), cultured for 24 h in RPMI-1640 medium, containing 1% penicillin and streptomycin (Capricorn Scientific GmbH) and 10% heat-inactivated fetal bovine serum (FBS) (Capricorn Scientific GmbH) and in an environment of 37°C and 5% CO_2_. For BMDC differentiation to bone marrow-derivate MΦ (BMDM), BMDC in the supernatant were centrifuged (250 ×  g; 10 min) and then cultured in RPMI-1640 (Capricorn Scientific GmbH) supplemented with 10% heat-inactivated FBS (Capricorn Scientific GmbH), 1% penicillin and streptomycin (Capricorn Scientific GmbH), and recombinant mouse GM-CSF (20 ng/ml) (BioLegend, San Diego, CA) and allowed to grow for 6 days. Incubation of BMDM of mice with IFN-γ and LPS for 24 h lead to the differentiation into BMDM1-MΦ, and incubation of BMDM with IL-4 and IL-13 for 24 h lead to the differentiation into BMDM2-MΦ.

#### 2.5.3. MΦ differentiation

The THP-1 M0-MΦ were incubated for 24 h with LPS (10 pg/ml, Sigma-Aldrich Co. LLC, St.Louis, USA) and IFN-γ (20 ng/ml, Provitro GmbH, Berlin) to differentiate into pro-inflammatory M1-MΦ [THP-1 M1-MФ]. For differentiation into anti-inflammatory M2-MΦ IL-4 (20 ng/ml, Provitro GmbH) and IL-13 (20 ng/ml, Provitro GmbH) were used for 24 h [THP-1 M2-MФ]. To control the successful differentiation of THP-1 into M1-/M2-MΦ, CCR7 and CCL17 mRNA expressions, as well as TNF-α, and IL-10 releases, were analyzed (6–8, 36, 37) (Additional File Figure S1). To examine the effect of PACAP38 (Bachem AG, Bubendorf, Schweiz), VPAC1-agonist [Ala11,22,28]VIP (Cat. Nr. 4040136, Bachem AG) or -antagonist [PG 97–269] (Cat. Nr. 4048647, Bachem AG), the THP-1 MФ were incubated for 24 h with PACAP38 (0.1 nM), [Ala11,22,28]VIP (0.1 nM) or PG 97–269 (10 nM) with or without 50 µg/ml oxLDL. PACAP38 concentration has been specified by Rasbach et al. (29).

### 2.6. LDL-oxidation

Native (n)LDL (Cell sciences, MA, USA) oxidation was performed as described by Galle and Wanner (38) and Steinbrecher (39). nLDL was suspended in endotoxin-free PBS without Ca^2+^, Mg^2+^ (LONZA, Ratingen, Germany) to a final concentration of 2 mg protein/ml, and dialyzed using Vivaspin™ 20—System (Thermo Fisher Scientific GmbH, Schwerte, Germany). The Vivaspin™ 20 centrifugal concentrator was sterilized with 70% EtOH for 10 min at 3,000 × g. Afterward, the Vivaspin™ 20 was washed with aqua dest (endotoxin-free). Then, nLDL suspended in PBS was transferred into the Vivaspin™ 20 and centrifuged for 20 min at 4,500 × g. Two washing steps with PBS were performed to remove ethylene diamine tetraacetic acid (EDTA) from the nLDL. CuSO_4_ was added to the EDTA-free nLDL and incubated overnight in the dark by constantly rotating. After 24 h, oxidation was stopped by adding EDTA (50 μM) and set in the dark for 1 h by continually rotating. After that, the oxLDL was washed three times with PBS. Subsequently, the oxLDL/PBS mixture was transferred by filtration through a 0.2 μm syringe filter to an endotoxin-free tube. The protein concentration was measured by Pierce™ BCA (bicinchoninic acid) Protein Assay (Thermo Scientific, Rockford, USA). We used different methods to determine the degree of oxidation: (1) trinitrobenzene sulfonic acid (TNBSA, Thermo Fisher Scientific GmbH), which measures free amino groups (40), (2) relative electrophoretic mobility (REM) by agarose gel electrophoresis and visualization by staining with Coomassie Blue (41), and 3. by spectrophotometric analysis (absorbance spectrum between 400 and 700 nm) (38).

### 2.7. Real-time quantitative (qRT) -PCR

The qRT-PCR was described before (34). QuantiTect primer assays (QIAGEN GmbH, Hilden, Germany) were used for qRT-PCR. All primers were purchased from QIAGEN GmbH (Hilden, Germany) (Table 3). Absorbance measurements at 260 nm and 280 nm (A260/A280 = 1.9–2.1) using a NanoDrop 8,000 spectrophotometer (Thermo Fisher Scientific GmbH) were used to determine RNA concentration and purity. Total RNA integrity was confirmed by lab-on-a-chip technology, using an RNA 6,000 NanoChip kit on an Agilent 2,100 Bioanalyzer (Stratagene-Agilent Technologies, Waldbronn, Germany). RNA was only used with an RNA Integrity Number (RIN) of ≥8.0. 1.0 μg of template RNA was used for cDNA synthesis. RNA was reverse transcribed using Oligo(dT)_12–18_ primer and 20 units of the Affinity-Script™ Multiple Temperature cDNA synthesis reverse transcriptase (Agilent Technologies) and 24 units of Ribo Lock™ RNAse inhibitor (Thermo Fisher Scientific) (1 h; 42°C). The cDNA (diluted 1:20) was amplified using the Brilliant III Ultra-Fast SYBR® Green qRT-PCR Master Mix (Stratagene-Agilent Technologies). Amplification and data analyses were performed using the Mx3005P™ qPCR System (Stratagene-Agilent Technologies). The data were analyzed using the relative standard curve method. For each sample, the relative quantity was calculated by linear regression analysis from the respective standard curves. The NormFinder software program was used to ascertain the most suitable reference gene (actin, beta, ACTB) to normalize the RNA input as described earlier (42).

**Table 3:** List of used primer for qRT-PCR. All primers were purchase by Qiagen GmbH, Hilden, Germany.

Primer	Amplicon length (bp)	Catalogue number	Ref. Sequence
Human			
ACTB	146	QT00095431	NM_001101
GAPDH	119	QT01192646	NM_002046
TBP	132	QT00000721	NM_001172085
CCR7	103	QT01666686	NM_001838
CCL17	76	QT00096866	NM_002987
Mouse			
VPAC1	149	QT00167160	NM_011703
ACTB	77	QT01136772	NM_007393
GAPDH	144	QT01658692	NM_008084
TBP	114	QT00198443	NM_013684
IL-10	103	QT00106169	NM_010548
TNF-α	112	QT00104006	NM_013693
IL-6	128	QT00098875	NM_031168
LOX-1	126	QT01759464	NM_138648
CD36	149	QT01058253	NM_001159555

### 2.8. Measurements of cytokine releases

The release of IL-6, IL-10, and TNF-α was quantified using an enzyme-linked immunosorbent assay (ELISA). According to the manufacturer's instructions, cytokines were determined in the culture medium using the assay DuoSet ELISA™ Development kit (R&D Systems Europe, Ltd., Abingdon, UK) (Table 4). The Capture Antibody was coated to a 96-well MaxiSorp™-ELISA Microplate (Nunc, San Diego, USA) and incubated overnight at room temperature. After the blocking, 100 µl samples or standards were added to the well. After the incubation with the detection antibody and streptavidin-horseradish peroxidase (HRP), the substrate solution [SigmaFast™ OPD (o-Phenylendiamin-dihydrochlorid), Sigma-Aldrich Chemie GmbH] was added to each well and incubated for 30 min in the dark. The reaction was stopped with 50 μl 3 M HCl and the optical density (OD) was measured at 490 nm and reference at 655 nm (OD 490/655) using a Sunrise microplate ELISA reader (Tecan Deutschland GmbH). The concentration of cytokines released into the medium was calculated by interpolation from the respective standard curves and normalized against the protein concentration.

**Table 4:** List of used kits.

Description	Company	Catalog number
Mouse IL-6 DuoSet ELISA	R&D Systems Europe	DY406
Mouse IL-10 DuoSet ELISA	R&D Systems Europe	DY417
Mouse TNF-α DuoSet ELISA	R&D Systems Europe	DY410
Human IL-10 DuoSet ELISA	R&D Systems Europe	DY217B
Human TNF-α DuoSet ELISA	R&D Systems Europe	DY210
Cholesterol/ Cholesteryl Ester Assay Kit—Quantitation	Abcam plc., Cambridge, UK	ab65359
Triglyceride Assay Kit—Quantification	Abcam plc., Cambridge, UK	ab65336

### 2.9. Determination of lipid droplets (LDs) by BODIPY™ 493/503

LDs are ubiquitous, dynamic organelles and serve as storage depots for neutral lipids, including triglycerides and cholesterol esters (43). The fluorescent neutral lipid dye 4,4-difluoro-1,3,5,7,8-pentamethyl-4-bora-3a,4a-diaza-s-indacene (BODIPY), which displays excitation (Ex)/emission (Em) maxima of 493/503 nm (Ex/Em = 493/503 nm) allows quantifying of fluorescence area containing neutral lipid. 5% PFA-fixed MΦ were stained with 4 µM BODIPY, (Thermo Fisher Scientific) for 15 min and visualized using a Nikon Eclipse Ti laser scanning microscope (Nikon GmbH, Düsseldorf, Germany). Cells were analyzed and fluorescence area was determined using ImageJ software (NIH). The mean BODIPY™ fluorescence area was normalized to nuclear DAPI counterstain for each sample.

### 2.10. OxLDL-uptake assay

THP-1 MΦ (3*10^5^ cells/ml) were differentiated as described above, treated with oxLDL-DyLight™ 488 (Cayman Chemical, Ann Arbor, MI, USA) 1:50 and incubated at 37°C for 24 h. After that, the culture medium was removed and Hoechst 33342 staining was performed (Cayman Chemical, Ann Arbor, MI, USA; 1:2,000) to determine the total nuclei fluorescence. Finally, we visualized cells on Laser-Scanning-Microscope Nikon Eclipse Ti (Nikon GmbH, Düsseldorf, Germany). According to the manufactureŕs instruction, the fluorometric measurement of treated MΦ was done at Ex/Em = 493/518 nm for and Ex/Em = 350/461 nm for Hoechst dye using the Cytation3 microplate reader (BioTek Instruments Inc., Winooski, VT, USA). The oxLDL-uptake was normalized against the total nuclei fluorescence stained with Hoechst 33342 (Cayman Chemical).

### 2.11. Total cholesterol assay of THP-1 Mф

Total cholesterol was determined using the Cholesterol/Cholesteryl Ester Quantitation Assay (Abcam plc., Cambridge, UK) (Table 4). THP-1 M1- /M2-MΦ (3*10^5^ cells/ml) treated in 6-well plates, as described above, were washed with ice-cold PBS and scraped in 150 μl PBS on ice. The suspension was transferred into a tube and centrifuged (10 min/250 × g/4°C). Thereafter, the supernatant was removed and the pellet was resuspended with a solution consisting of chloroform, isopropanol and NP40 (7:11:0.1). An additional centrifugation step was performed (10 min/15,000 × g/4°C). Subsequently, all phases of the supernatant were transferred to a fresh tube and air-dried overnight under the hood to remove the chloroform. The sample was then dissolved in assay buffer. 25 μl of the sample or a standard serial dilution were pipetted into a 96-well plate, refilled to 50 μl with the total cholesterol reaction mix and incubated for 1 h at RT in the dark. The total cholesterol concentration was measured with a microplate reader (Tecan) at OD595/655 nm and determined using a standard curve.

### 2.12. Triglyceride quantification assay of THP-1 Mф

Triglycerides were determined using Triglyceride Quantification Colorimetric Assay Kit (Abcam plc.) (Table 4) according to manufacturer's instructions. The M1-/M2-MΦ (3*10^5^ cells/ml) treated in 6-well plates, as described above, were homogenized in 5% NP-40/ddH_2_O and heated at 92°C for 5 min. After that, the samples were centrifuged by 13,000 × g and the supernatant was diluted 1:10 with aqua dest. Additionally, lipase was added to the samples and incubated for 20 min RT. The master mix of Triglyceride Assay Buffer, Triglyceride Probe and Triglyceride Enzyme Mix was prepared according to the manufacturer's instructions, added to the samples and incubated in the dark for 60 min. The fluorometric measurements were done at Ex/Em = 535/585 nm using the Cytation3 microplate reader (BioTek Instruments Inc.). Triglyceride concentration was determined by using a standard curve.

### 2.13. SDS page and western blot

THP-1 M1-/M2-MΦ (3*10^5^ cells/ml), treated in 6-well plates as described above, were washed in ice-cold PBS and lysed with radioimmunoprecipitation buffer (RIPA) pH 7.5 (Cell Signaling Technology, Frankfurt, Germany) containing protease/phosphatase inhibitor cocktail (Cell Signaling Technology). Protein concentrations were determined spectrophotometrically using the Pierce™ BCA Protein Assay (Thermo Scientific). 30 µg of proteins were loaded onto a NuPAGE® Novex® 4%–12% Bis-Tris gel (Life Technologies GmbH, Darmstadt, Germany). By wet blot, proteins were transferred to a 0.45 μm nitrocellulose membrane (Millipore, Billerica, MA, USA). Primary antibodies (Table 2) were incubated overnight at 4°C in blocking buffer (5% nonfat milk in Tris-buffered saline with Tween20). Incubation of the 2nd antibody [donkey anti-rabbit IgG, HRP-linked F(ab’)2-fragment; Table 2] was performed at room temperature for 1 h at room temperature. The peroxidase reaction was visualized using AceGlow chemiluminescent substrate (PEQLAB GmbH, Erlangen, Germany) and documented using the Fusion-SL Advance™ Imaging System (PEQLAB GmbH) according to the instructions in the manual. The intensity of specific Western blot bands was quantified using ImageJ software from the National Institutes of Health (Bethesda, USA). Normalization was performed against α-tubulin.

### 2.14. Statistical analyses

Statistical analyses were performed using SigmaPlot 12 (Systat Software Inc., USA). After testing for normality (by Shapiro-Wilk), the unpaired Student's t-test or one-way analysis of variance (ANOVA) was performed. Data are reported as mean + standard deviation (SD), *p* ≤ 0.05 was considered statistically significant.

## 3. Results

### 3.1. PACAP deficiency affects luminal stenosis and body weight

**Figure 1:**
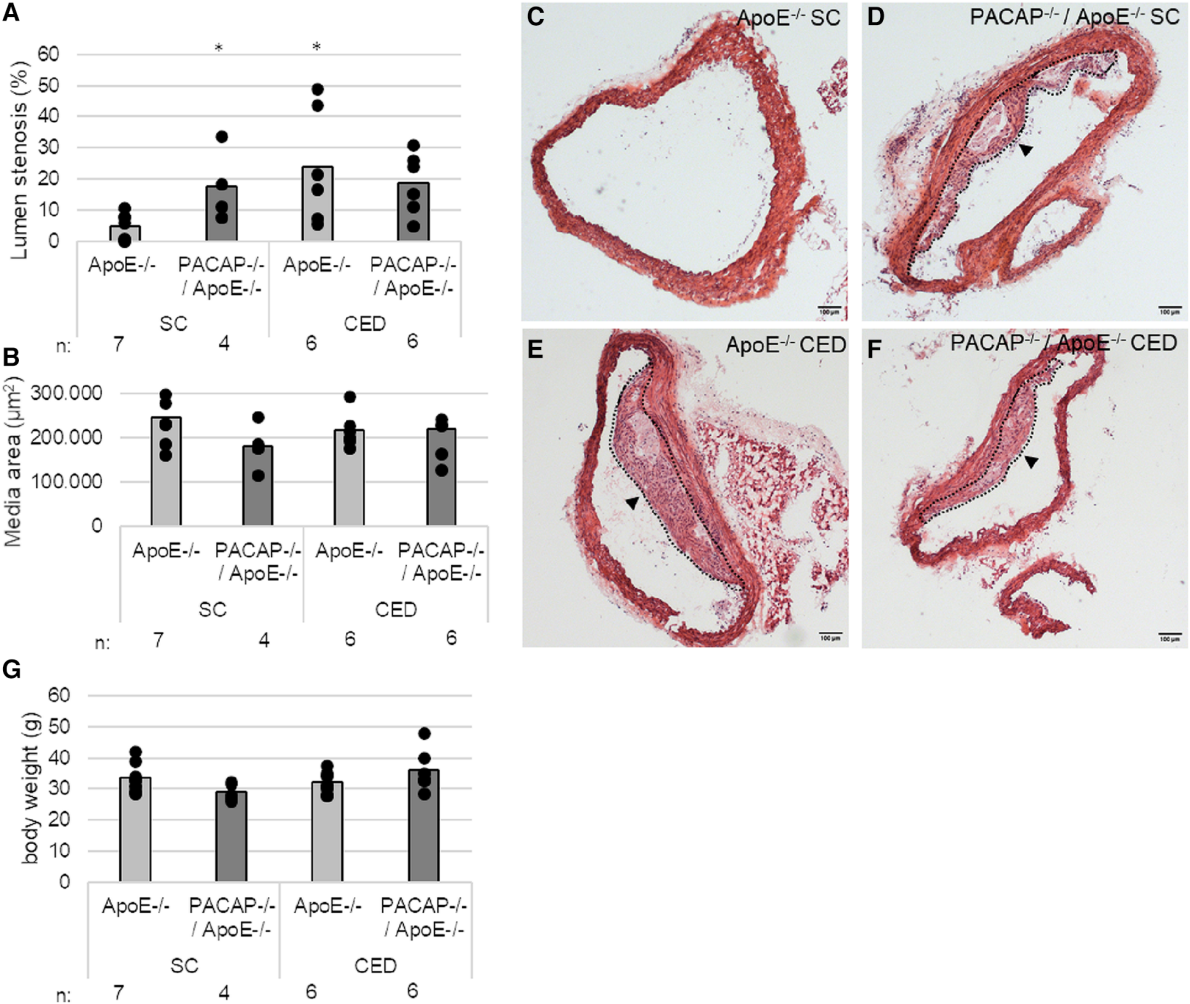
Effects of PACAP deficiency on the aortic arch (AA) lumen stenosis and body weight in ApoE^−/−^ and PACAP^−/−^/ApoE^−/−^ mice after 30 weeks of SC or 20 weeks of CED. (**A**) Lumen stenosis (%) and (**B**) media area (*μ*m^2^) were measured in AA by computer-assisted morphometry. (**C–F**) representative HE-stained histological cross sections of AA from PACAP^−/−^/ApoE^−/−^ and ApoE^−/−^ mice. Black arrowhead: atherosclerotic plaque; broken line: outline of the atherosclerotic plaque; scale bar: 100 μm. (**G**) body weight in g. *p ≤ 0.05 vs. ApoE^−/−^ mice after 30 weeks of SC. SC, standard diet; CED, cholesterol-enriched diet; n, number of experimental animals.

Given the increased lumen stenosis in PACAP^−/−^/ApoE^−/−^ mice after 30 weeks of standard chow (SC), which we have demonstrated in this study (3.8-fold; *p* = 0.024) (Figures 1A,C–F) as well as in a recent study (29), we also investigated the effects on media thickness and body weight of PACAP deficiency in ApoE^−/−^ mice. After 10 weeks of SC following 20 weeks of CED, lumen stenosis increased 3.8-fold in ApoE^−/−^ mice only (*p* = 0.021), whereas no change in lumen stenosis was observed in PACAP^−/−^/ApoE^−/−^ mice after this observation period (Figures 1A,E,F). Media thickness in ApoE^−/−^ and PACAP^−/−^/ApoE^−/−^ at 30 weeks of SC or 20 weeks of CED remained unaffected (Figure 1B). Body weight in PACAP^−/−^/ApoE^−/−^ mice compared to ApoE^−/−^ mice was decreased by 14% (*p* = 0.056) after 30 weeks of SC (Figure 1G). After 20 weeks of CED, body weight increased by 25% (*p* = 0.058) in PACAP^−/−^/ApoE^−/−^ mice (Figure 1G).

### 3.2. Expression of VPAC1 receptors and Mф markers in atherosclerotic plaque

**Figure 2:**
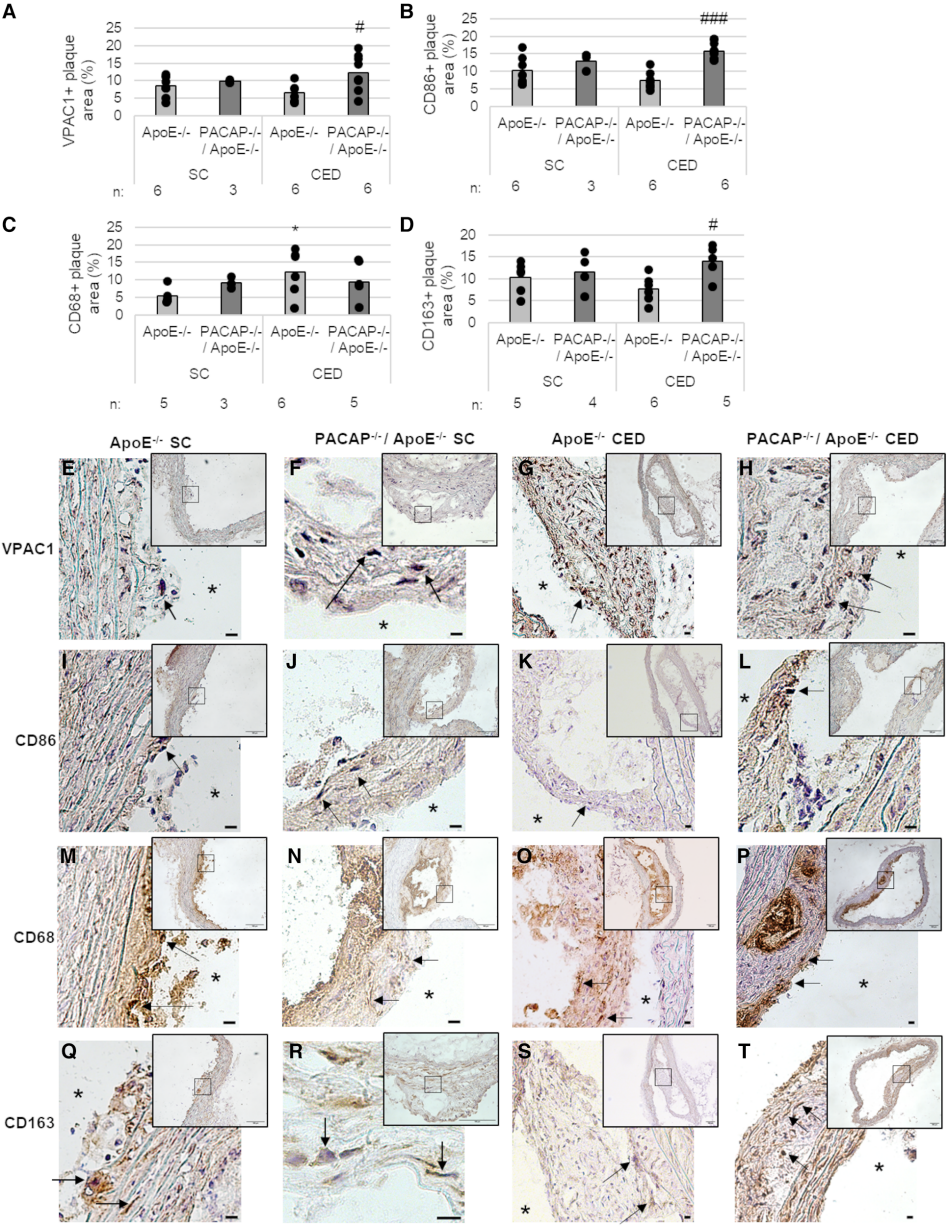
Effects of PACAP deficiency on the distribution and amount of different MΦ subtypes of atherosclerotic plaques in the aortic arch. Immunohistomorphometric analyses of atherosclerotic lesions in ApoE^−/−^ and PACAP^−/−^/ApoE^−/−^ mice after SC or CED. Analyses of VPAC1 (**A**), CD86 (**B**), CD68 (**C**), and CD163 (**D**) on cryosections of the aortic arch and representative photomicrographs (**E–T**) are shown. The graph bar is given as the mean. Black dots represent single measurement. (*n* = 3–8). *p ≤ 0.05 vs. ApoE^−/−^ after 30 weeks of SC; ^#^*p* ≤ 0.05; ^###^*p* ≤ 0.001 vs. ApoE^−/−^ after 20 weeks of CED. SC, standard diet; CED, cholesterol-enriched diet; n, number of experimental animals; Black arrows, positive reaction; asterisks, vessel lumen. Scale bar = 10 μm; Overview screen scale bar = 100 µm.

We immunohistochemically determined the expression of VPAC1 in atherosclerotic plaques of the aortic arch of PACAP^−/−^/ApoE^−/−^ and ApoE^−/−^ mice after 30 weeks of SC and 20 weeks of CED (Figures 2A,E–H), because it has been shown that the VPAC1 agonist (Ala11,22,28)-VIP aggravated early atherosclerosis in hypercholesterolemic ApoE^−/−^ mice (44). In PACAP^−/−^/ApoE^−/−^ mice after 20 weeks of CED, VPAC1-immunoreactive plaque area was increased by 5.78% (*p* = 0.033) compared with ApoE^−/−^ mice (Figure 2A). VPAC1-positive cells were found to be located in the fibrotic cap and shoulder regions, as well as in the media (Figures 2E–H).

We used different MФ markers (CD86, CD68, CD163) to measure the respective immunoreactive atherosclerotic plaque areas in the aortic arch of ApoE^−/−^ and PACAP^−/−^/ApoE^−/−^ mice (Figures 2B–D,I–T). After 20 weeks of CED, PACAP^−/−^/ApoE^−/−^ mice revealed an 8.45% (*p* < 0.001) higher CD86-immunoreactive area than ApoE^−/−^ mice (Figure 2B). Moreover, after 30 weeks SC, PACAP^−/−^/ApoE^−/−^ mice showed a 3.82% (*p* = 0.053) increased CD68-immunoreactive area compared to ApoE^−/−^ (Figure 2C). Additionally, ApoE^−/−^ mice fed for 20 weeks with CED revealed 6.8% elevated CD68-immunoreactive area compared to ApoE^−/−^ after SC for 30 weeks (Figure 2C). After 20 weeks CED, PACAP^−/−^/ApoE^−/−^ mice showed a significant (*p* = 0.017) 7.44% higher CD163-immunoreactive area than ApoE^−/−^ mice (Figure 2D). ApoE^−/−^ mice fed for 20 weeks with CED had a 3.76% (*p* = 0.07) lower CD163-immunoreactive area than ApoE^−/−^ mice after SC (Figure 2D). However, CD86-positive cells were ubiquitously found in atherosclerotic plaques predominantly in the fibrotic cap (Figures 2I–L). CD68-immunoreactive areas were localized around the necrotic core and shoulder regions (Figures 2M–P), whereas the CD163-positive cells were predominantly found in the media, and fibrotic cap regions of the atherosclerotic plaque (Figures 2Q–T).

Based on our findings that VPAC1 immunoreactivity was found in regions where CD163-positive cells were localized, we further aimed to investigate *in vitro* different MΦ subtypes and the role of VPAC1 and PACAP concerning inflammatory processes and lipid homeostasis.

### 3.3. Expression of VPAC1 receptors in bone marrow-derived MΦ subtypes

**Figure 3:**
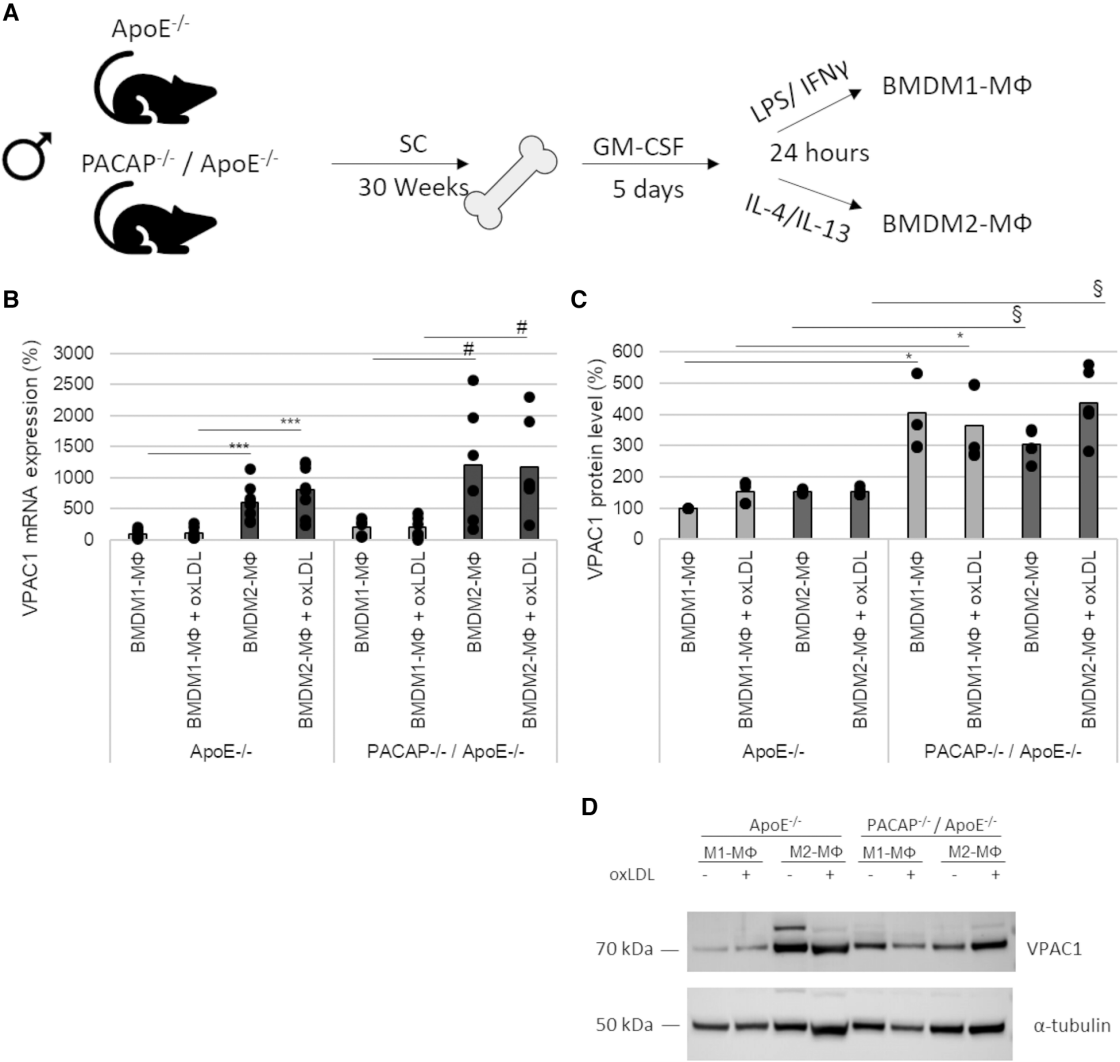
Expression of VPAC1 receptor in ApoE^−/−^ and PACAP^−/−^/ApoE^−/−^ BMDM1- and BMDM2-MΦ. (**A**) Experimental setup for the extraction and differentiation of BMDM1- and BMDM2-MΦ from femur cells. SC, standard diet; GM-CSF, Granulocyte-macrophage colony-stimulating factor. (**B**) VPAC1 mRNA expression was determined by qRT-PCR, normalized by GAPDH. ApoE^−/−^ BMDM1-MΦ was set to 100%; *n* = 6. (**C**) VPAC1 protein values were determined by western blot, normalized against *α*-tubulin, and analyzed by Fiji software. Bar graph shows the mean value. The black dots show the single measurement (*n* = 6). (**D**) Representative western blots of VPAC1 and *α*-tubulin are shown. *p ≤ 0.05, ***p ≤ 0.001 vs. ApoE^−/−^ BMDM1-MΦ; ^#^*p* ≤ 0.05 vs. PACAP^−/−^/ApoE^−/−^ BMDM1-MΦ; ^§^*p* ≤ 0.05 vs. ApoE^−/−^ BMDM2-MΦ.

To determine whether VPAC1 receptors are expressed in different MΦ subtypes, BMDM were isolated from the femur of ApoE^−/−^ and PACAP^−/−^/ApoE^−/−^ mice and differentiated into BMDM1- and BMDM2-MΦ (Figure 3A). The mRNA and protein expressions of VPAC1 were measured after incubation with/without oxLDL. VPAC1 mRNA expression was increased 5-fold (*p* = 0.014) in ApoE^−/−^ BMDM2-MΦ and 6-fold (*p* = 0.044) in PACAP^−/−^/ApoE^−/−^ BMDM2-MΦ compared to ApoE^−/−^ and PACAP^−/−^/ApoE^−/−^ BMDM1-MΦ, respectively (Figure 3B). Interestingly, PACAP^−/−^/ApoE^−/−^ BMDM1- and BMDM2-MΦ showed a 4-fold (*p* = 0.045) and 2-fold (*p* = 0.045) higher VPAC-1 protein level compared to ApoE^−/−^ BMDM (Figures 3C,D). In either BMDM-subtypes of ApoE^−/−^ or PACAP^−/−^/ApoE^−/−^ mice incubation with oxLDL for 24 h did not affect VPAC1 mRNA expression or VPAC1 protein level (Figures 3B–D).

### 3.4. Effect of PACAP-deficiency on inflammation in BMDM1- and BMDM2-MΦ

In the present study, we analyzed the anti-inflammatory properties of PACAP in classically activated (inflammatory) M1-MΦ and anti-inflammatory M2-MΦ *in vitro*.

**Figure 4:**
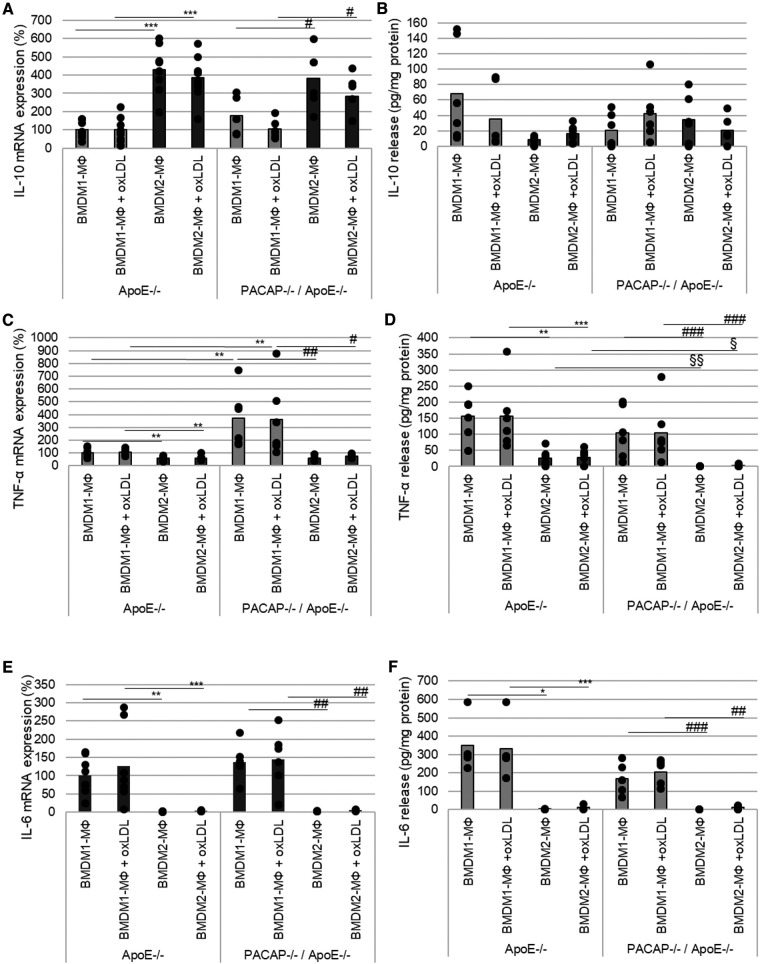
PACAP deficiency influenced TNF-α mRNA expression and release in ApoE^−/−^ BMDM. The mRNA expression of IL-10 (**A**), TNF-α (**C**) and IL-6 (**E**) were determined by qRT-PCR and normalized against GAPDH. ApoE^−/−^ BMDM1-MΦ was set 100%. (*n* = 5–8). IL-10 (**B**), TNF-α (**D**) and IL-6 (**F**) release (pg/ml) of BMDM1- and BMDM2-MΦ were determined using ELISA [OD490/655]. Bar graph shows the mean value. The black dots show the single measurement (*n* = 13). *p ≤ 0.05, **p ≤ 0.01, ***p ≤ 0.001 vs. ApoE^−/−^ BMDM1-MΦ; ^§^*p* ≤ 0.05, ^§§^*p* ≤ 0.01 vs. ApoE^−/−^ BMDM2-MΦ; ^#^*p* ≤ 0.05, ^###^*p* ≤ 0.01, ^###^*p* ≤ 0.001 vs. PACAP^−/−^/ApoE^−/−^ BMDM1-MΦ.

IL-10 mRNA expression was significantly increased in ApoE^−/−^ BMDM2-MΦ and PACAP^−/−^/ApoE^−/−^ BMDM2-MΦ compared with the corresponding BMDM1-MΦ (ApoE^−/−^ BMDM2-MΦ with medium: 330%; with oxLDL: 285%; PACAP^−/−^/ApoE^−/−^ BMDM2-MΦ with medium: 205%; with oxLDL: 178%; *p* ≤ 0.05) (Figure 4A). PACAP deficiency or treatment with oxLDL did not affect IL-10 mRNA expression in either BMDM-subtype (Figure 4A). Additionally, the IL-10 release is independent of PACAP, oxLDL and BMDM-subtype (Figure 4B).

In BMDM2-MΦ, TNF-α mRNA expression was significantly lower than in BMDM1-MΦ (ApoE^−/−^ BMDM2-MΦ with medium: 41%; with oxLDL: 44%; PACAP^−/−^/ApoE^−/−^ BMDM2-MΦ with medium: 312%; with oxLDL: 285%; *p* ≤ 0.008) (Figure 4C). Moreover, in BMDM1-MΦ of PACAP^−/−^/ApoE^−/−^ mice TNF-α mRNA expression was increased by 269% (*p* = 0.005) without oxLDL addition and by 256% (*p* = 0.014) with oxLDL, respectively (Figure 4C). In general, the TNF-α release was significantly lower in BMDM2-MΦ than in BMDM1-MΦ (ApoE^−/−^ BMDM 2-MΦ with medium: 84%; with oxLDL: 84%; PACAP^−/−^/ApoE^−/−^ BMDM2-MΦ with medium: 100%; with oxLDL: 98%; *p* ≤ 0.009) (Figure 4D). PACAP^−/−^/ApoE^−/−^ BMDM2-MΦ showed significantly decreased TNF-α release compared to ApoE^−/−^ BMDM2-MΦ (PACAP^−/−^/ApoE^−/−^ BMDM2-MΦ with medium: 100%; with oxLDL: 93%; *p* ≤ 0.002) independent of oxLDL-treatment (Figure 4D).

IL-6 mRNA expression was significantly reduced in BMDM2-MΦ compared with BMDM1-MΦ (ApoE^−/−^ BMDM2-MΦ with medium: 98%; with oxLDL: 123%; PACAP^−/−^/ApoE^−/−^ BMDM2-MΦ with medium: 135%; with oxLDL: 140%) (Figure 4E). PACAP deficiency or incubation with oxLDL had no effect on IL-6 mRNA expression in either BMDM-subtype (Figure 4E). IL-6 release was significantly decreased in BMDM2-MΦ compared with BMDM1-MΦ (ApoE^−/−^ BMDM2-MΦ with medium: 99%; with oxLDL: 96%; PACAP^−/−^/ApoE^−/−^ BMDM2-MΦ with medium: 100%; with oxLDL: 94%; *p* ≤ 0.02) independent from PACAP or oxLDL-treatment (Figure 4F).

### 3.5. Effect of PACAP-deficiency on lipid homeostasis in BMDM1- and BMDM2-MΦ

**Figure 5:**
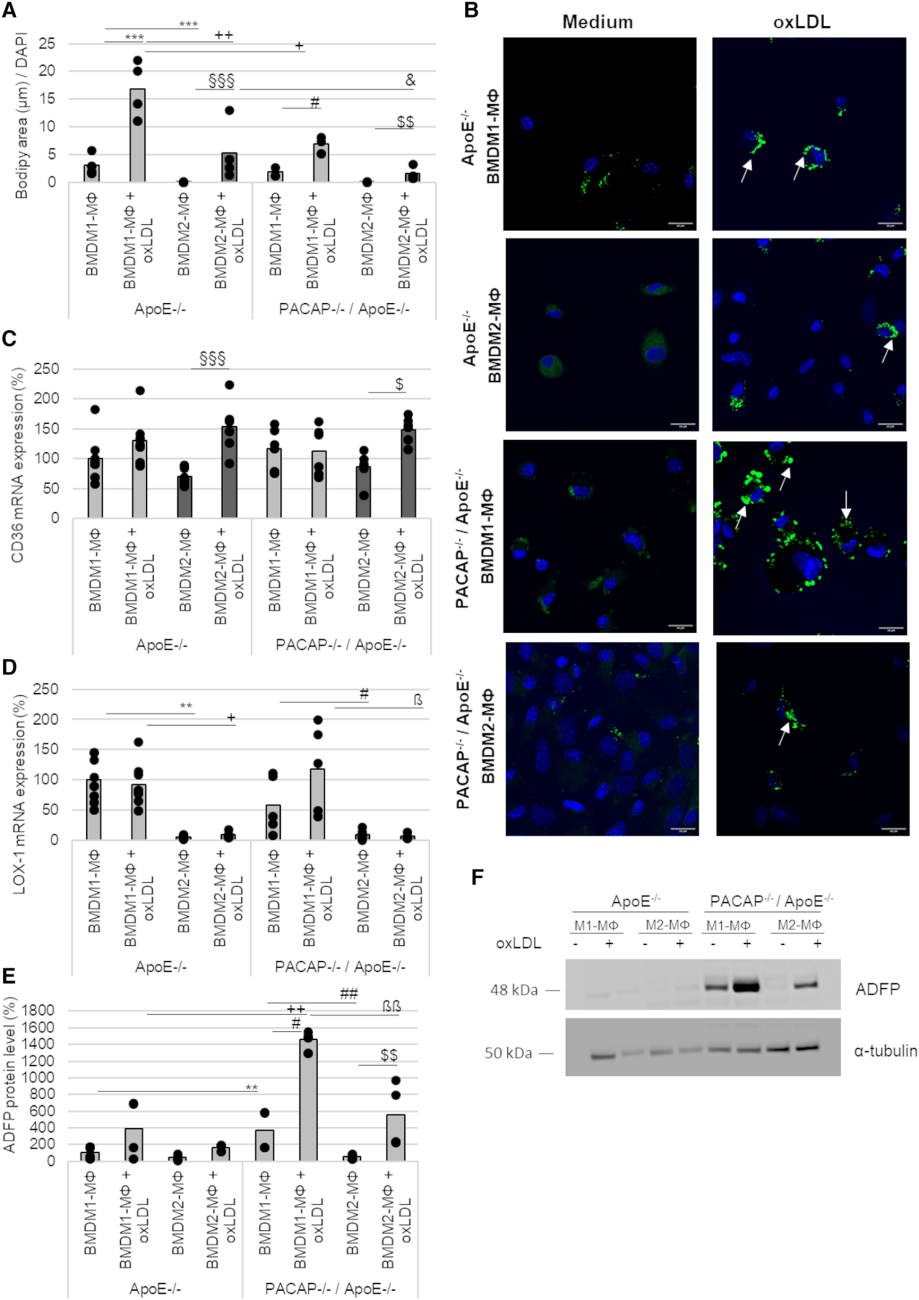
Lipid accumulation and expression of scavenger receptors in ApoE^−/−^ and PACAP^−/−^/ApoE^−/−^ BMDM1- and BMDM2-MΦ after oxLDL incubation. (**A**) The fluorescence area of BODIPY™ 493/503 were analyzed by Fiji software and normalized against DAPI (total cell number). Bar graph shows the mean value. The black dots show the single measurement (*n* = 4). (**B**) The representative images show DAPI staining (blue) with BODIPY™ 493/503 (green) by confocal laser scanning microscopy (Nikon Eclipse). White arrows indicate lipid droplets (LDs). Scale bars = 20 µm. mRNA expression of CD36 (**C**) and LOX-1 (**D**) were studied by qRT-PCR and normalized against GAPDH expression. ApoE^−/−^ BMDM1-MΦ was set to 100%. Bar graph shows the mean value. The black dots show the single measurement (*n* = 5–8). (**E**) ADFP protein values were determined by western blot, normalized against *α*-tubulin, and analyzed by ImageJ. ApoE^−/−^ BMDM1-MΦ was set to 100%. Bar graph shows the mean value. The black dots show the single measurement (*n* = 4). (**F**) Representative western blots of ADFP and *α*-tubulin are shown. **p ≤ 0.01, ***p ≤ 0.001 vs. ApoE^−/−^ BMDM1-MΦ; ^+^*p* ≤ 0.05, ^++^*p* ≤ 0.01 vs. ApoE^−/−^ BMDM1-MΦ with oxLDL; ^§§§^*p* ≤ 0.001 vs. ApoE^−/−^ BMDM2-MΦ; ^&^*p* ≤ 0.05 vs. ApoE^−/−^ BMDM2-MΦ with oxLDL; ^#^*p* ≤ 0.05 vs. PACAP^−/−^/ApoE^−/−^ BMDM1-MΦ; ^$^*p* ≤ 0.05, ^$$^*p* ≤ 0.01 vs. PACAP^−/−^/ApoE^−/−^ BMDM2-MΦ; ^ß^*p* ≤ 0.05, ^ßß^*p* ≤ 0.01 vs. PACAP^−/−^/ApoE^−/−^ BMDM1-MΦ with oxLDL.

MΦ actively participate in lipoprotein-uptake via scavenger receptors and thereby develop into foam cells. Due to its nonpolar structure, long-wavelength absorption, and fluorescence, BODIPY™ (Ex/Em = 493/503 nm) was used as a dye to detect intracellular triglycerides (TAGs). Thereby, 24 h oxLDL incubation significantly increased BODIPY™ fluorescence area [normalized to DAPI fluorescence (Ex/Em = 359/457 nm)] by 6-fold (*p* ≤ 0.001) in ApoE^−/−^ BMDM1- and by 88-fold (*p* ≤ 0.001) in ApoE^−/−^ BMDM2-MΦ compared to BMDM incubated with medium alone (∼ medium control) (Figures 5A,B). Likewise, oxLDL treatment of PACAP^−/−^/ApoE^−/−^ BMDM1- and BMDM2-MΦ showed 3-fold (*p* ≤ 0.05) increased BODIPY™ fluorescence area compared to corresponding BMDM incubated in medium alone (∼control) (Figures 5A,B). However, interestingly, PACAP^−/−^/ApoE^−/−^ BMDM revealed a significantly lower oxLDL-induced BODIPY™ fluorescence area by 2-fold (*p* = 0.026) in BMDM1- and by 3-fold (*p* = 0.021) in BMDM2-MΦ compared to the corresponding ApoE^−/−^ BMDM (Figures 5A,B). Additionally, investigations of the cell morphology by fluorescence images revealed that PACAP^−/−^/ApoE^−/−^ BMDM1-MΦ incubated with oxLDL exhibited a foam cell-like morphology compared with the oxLDL-treated ApoE^−/−^ BMDM1-MΦ (Figure 5B): (1) the cells were larger and (2) the lipid droplets (LDs) were more concentrated at the cell edge (Figure 5B). The LDs in oxLDL-treated ApoE^−/−^ BMDM1-MΦ were predominantly localized in the periphery of the nucleus (Figure 5B).

The scavenger receptors CD36 and oxLDL receptor-1 (LOX-1) mRNA expression were determined in BMDM1-/M2-MΦ (Figures 5C,D). CD36 mRNA expression was increased by 84% (*p* ≤ 0.001) in ApoE^−/−^ BMDM2-MΦ and by 63% (*p* = 0.044) in PACAP^−/−^/ApoE^−/−^ BMDM2-MФ after oxLDL-treatment (Figure 5C). LOX-1 mRNA expression was reduced in ApoE^−/−^ BMDM2-MΦ (with medium: 95%; with oxLDL: 84%; *p* ≤ 0.032) and PACAP^−/−^/ApoE^−/−^ BMDM2-MΦ (with medium: 52%; with oxLDL: 111%; *p* ≤ 0. 032) compared to the corresponding BMDM1-MΦ (Figure 5D).

PACAP^−/−^/ApoE^−/−^ BMDM1-MΦ showed higher ADFP protein levels compared to ApoE^−/−^ BMDM1-MΦ (with medium: 3-fold; with oxLDL: 3-fold; *p* = 0.002) (Figures 5E,F). Additionally, PACAP^−/−^/ApoE^−/−^ BMDM1-MΦ showed a 7-fold (*p* = 0.017) increase in ADFP levels compared to PACAP^−/−^/ApoE^−/−^ BMDM2-MΦ (Figures 5E,G). Treatment with oxLDL resulted in a 4-fold (*p* = 0.002) increase in the amount of ADFP in PACAP^−/−^/ApoE^−/−^ BMDM1- and a 10-fold increase in the amount of ADFP in PACAP^−/−^/ApoE^−/−^ BMDM2-MΦ (*p* = 0.004) compared to the medium control (Figures 5E,F).

### 3.6. Effect of PACAP38 and VPAC1 on lipid accumulation in human THP-1 M1- and M2-MΦ

**Figure 6:**
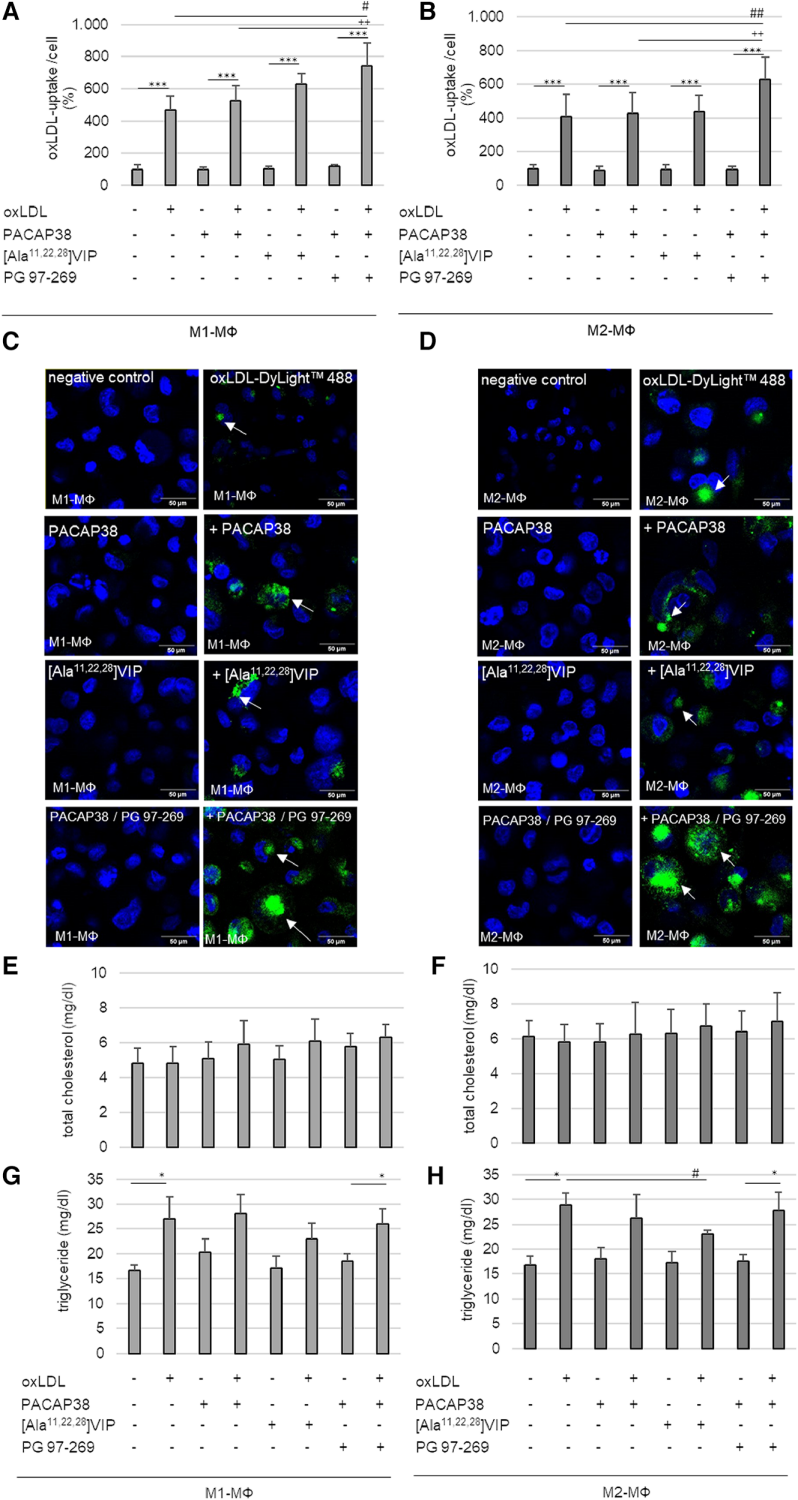
oxLDL-uptake and lipid homeostasis in human PMA-differentiated THP-1 M1- and M2-MΦ, were treated with oxLDL and PACAP38, VPAC1-agonist [Ala11,22,28]VIP or -antagonist PG 97–269/PACAP38. Percentage of oxLDL-uptake per cell by (**A**) M1- and (**B**) M2-MΦ. The fluorescence intensity of oxLDL-DyLight™488 was measured by Cytation™3 imaging reader (Ex/Em = 493/518) and normalized against Hoechst DNA nuclear counterstain (as total cell number). Negative (medium alone) control was set to 100%. Results are expressed as means + SD (*n* = 5). (**C,D**) The representative images show Hoechst staining with or without oxLDL-DyLight™488 (green) uptake (white arrows) by confocal laser scanning microscopy (Nikon Eclipse). Scale bars = 50 µm. Total cholesterol contents were measured with an ELISA reader (OD 595/655 nm) in (**E**) M1- and (**F**) M2-MΦ. Data in mg/dl are expressed as means + SD (*n* = 4). The triglyceride content in (**G**) M1- and (**H**) M2-MΦ was fluorometric measured at (Ex/Em = 535/585) using the Cytation3 imaging reader. Data in mg/dl are expressed as means + SD (*n* = 4). *p ≤ 0.05, ***p ≤ 0.001 vs. without oxLDL; ^#^*p* ≤ 0.05, ^##^*p* ≤ 0.01 vs. oxLDL; ^++^*p* ≤ 0.01 vs. oxLDL/PACAP38.

Concerning the data from BMDM, we analyzed the percentage of oxLDL-uptake per cell in human M1- and M2-MΦ. For this purpose, we used the PMA-differentiated human THP-1, a cell line routinely used in atherosclerosis research. The uptake of oxLDL could be detected in all THP-1 M1-and M2-MΦ based on the fluorescence intensity of oxLDL-DyLight™488(Ex/Em = 493/518) compared with MΦ incubated in medium alone (∼control) (M1-MΦ: with oxLDL: 4-fold; with PACAP38/oxLDL: 4-fold; with [Ala11,22,28]VIP/oxLDL: 6-fold; with PG 97–269/PACAP38/oxLDL: 6-fold; M2-MΦ: with oxLDL: 3-fold; with PACAP38/oxLDL: 3-fold; with [Ala11,22,28]VIP/oxLDL: 3-fold; with PG 97–269/PACAP38/oxLDL: 5-fold; *p* ≤ 0.001) (Figures 6A–D). To investigate the function of VPAC1, THP-1 M1- and M2-MΦ were additionally treated with the VPAC1-antagonist PG 97–269 or the VPAC1 agonist [Ala11,22,28]VIP. Our data show that incubation with the VPAC1-antagonist PG 97–269 in combination with PACAP38 significantly increased the uptake of oxLDL-DyLight™488 by 2-fold (*p* ≤ 0.004) in THP-1 M1- and M2-MΦ compared to PACAP38/oxLDL-DyLight™488 (Figures 6A–D).

Analyses of total cholesterol and triglyceride levels in human THP-1 MΦ should provide information, whether PACAP38 or VPAC1 plays a role in lipid homeostasis in THP-1 M1-/M2-MΦ. However, neither oxLDL with or without PACAP38 nor [Ala11,22,28]VIP altered intracellular total cholesterol concentrations in either MΦ subtype (Figures 6E,F). Analyses of intracellular triglyceride content showed that incubation of both THP-1 MΦ subtypes with oxLDL resulted in an increase in intracellular triglyceride (M1-MΦ: 39%; M2-MΦ: 42%; *p* ≤ 0.03) compared to cells incubated in medium alone (∼control) (Figures 6G,H). Moreover, co-incubation of THP-1 MΦ with oxLDL as well as with the VPAC1 antagonist PG 97–269/PACAP38 significantly increased triglyceride concentration in both MΦ subtypes (M1-MΦ: 29%; M2-MΦ: 37%; *p* ≤ 0.034) compared to THP-1 MΦ incubated with PG 97–269/PACAP38 without oxLDL (Figures 6G,H). On the other hand, co-incubation of THP-1 MΦ with oxLDL as well as with PACAP38 or with [Ala11,22,28]VIP in both MΦ subtypes did not result in an increase in triglyceride concentration compared with PACAP38 or [Ala11,22,28]VIP alone (Figures 6G,H). In THP-1 M2-MΦ, even co-incubation of oxLDL with the VPAC1 agonist [Ala11,22,28]VIP significantly (*p* = 0.026) lowered intracellular triglyceride concentration by 20% compared to oxLDL alone (Figure 6H).

## 4. Discussion

The main novel findings of our studies were that PACAP deficiency in ApoE^−/−^ mice promoted the development of atherosclerotic plaques in the aortic arch under SC (30 weeks) and led to an increase of VPAC1-immunoreactive area after 20 weeks of CED. These data confirm, previously published findings that PACAP deficiency aggravates atherosclerosis in the brachiocephalic trunk in ApoE^−/−^ mice after 30 weeks of SC (29). To VPAC1, it is known that VIP and PACAP can bind to the receptor with equal affinity (Kd = 1.0 nM) (45). VIP is known to regulate cholesterol efflux in MΦ and to reduce foam cell formation (46). Further studies showed that systemic VIP treatment reduced the number and size of atherosclerotic plaques in the carotid artery, aorta, and sinus in hypercholesterolemic mice (46). Therefore, the presence of VPAC1 in atherosclerotic plaques suggests the possibility that PACAP/VIP signaling plays a critical role in atherogenesis.

In addition to increased lumen stenosis, weight loss in PACAP^−/−^/ApoE^−/−^ mice after 30 weeks of SC was also verified within this study. This is consistent with the previous research by Rasbach et al. (29). PACAP neurons of the paraventricular neuron are thought to promote appetite through synaptic connections of NPY/AgRP neurons (47). Loss of this signaling through PACAP deficiency may explain the reports of hypoinsulinemia, decreased adiposity, lower body weight, and increased insulin sensitivity in PACAP-null transgenic mice (48).

In view of inflammatory processes, VPAC1 mediates the immunomodulatory properties of PACAP and VIP (49). After demonstrating that PACAP deficiency in ApoE^−/−^ mice leads to an increase in VPAC1-immunoreactive area in the plaque after 20 weeks of CED feeding, we examined the differences in MΦ subtypes in plaque between PACAP^−/−^/ApoE^−/−^ and ApoE^−/−^ mice. In this context, we were able to show, that after 20 weeks of CED PACAP^−/−^/ApoE^−/−^ mice led to an increased CD86- and CD163- immunoreactive areas compared to ApoE^−/−^ mice. CD86 located on the surface of antigen-presenting cells, including classical M1- and M2b-MΦ provides costimulatory signals necessary for T cell activation and survival (50). Activated T cells proliferate, release effector molecules (IFN-*γ*, IL-2), and enhance cytotoxic activity (50, 51). CD163 is a monocyte/macrophage-specific marker for M2-MΦ expressed primarily on cells with strong anti-inflammatory potential (52). Our data show that CD163-positive cells were most frequently localized on the cap of the plaque and in the media. Interestingly, VPAC1-positive cells were also predominantly located on the cap of atherosclerotic plaques and the subendothelial space. Additionally, after 30 weeks of SC, the CD68-immunoreactive area was more pronounced in PACAP^−/−^/ApoE^−/−^ mice compared with ApoE^−/−^ mice and was predominantly located in the necrotic core and shoulder region. CD68 is generally considered to be a selective marker and a lysosomal protein that is highly expressed by human monocytes and tissue MΦ (50). However, several immunohistochemical studies showed that CD68 antibodies can also react with other hematopoietic and nonhematopoietic cell types (53). Accordingly, it is generally known that pro-inflammatory M1-MΦ are pro-atherogenic, whereas the anti-inflammatory M2-MΦ subtype appears to be anti-atherogenic (15, 16). The recruited MΦ in the subendothelial space represent several different polarized phenotypes, which have multiple implications for lesion development and progression (54). M1-MΦ exist in symptomatic plaques and are predominantly localized in shoulder regions, whereas M2-MΦ are present in stable, asymptomatic plaques, predominantly localized in the adventitia and fibrotic cap of plaques (9, 10). M1-MΦ characterize progressive lesions, whilst regressing plaques are enriched in M2-MΦ (55). However, M1- and M2-MΦ contribute to diverse stages of plaque development and are localized in distinct morphological areas within atherosclerotic lesions. Our data show that the proportion of anti-inflammatory markers, like CD163 is increased in PACAP-deficient ApoE^−/−^ mice, suggesting a stabilization of the atherosclerotic plaque. Therefore, we further investigated M1- and M2-MΦ in the context of PACAP and the receptor VPAC1.

For this purpose, BMDM from ApoE^−/−^ and PACAP^−/−^/ApoE^−/−^ mice were differentiated into M1-MФ using LPS/IFNγ or into M2-MФ using IL-4/IL-13. Stein et al. (56) were the first to describe an alternative MΦ subtype induced by IL-4 and characterized by high mannose receptor expression (56). Moreover, the proinflammatory M1-MΦ phenotype is known to release proinflammatory cytokines (IL-1β, TNF-α), whereas the M2-MΦ phenotype releases factors such as transforming growth factor (TGF) β or IL-10, which limit the inflammatory process (54). In this study, we successfully differentiated ApoE^−/−^ and PACAP^−/−^/ApoE^−/−^ BMDM into BMDM1-MФ and BMDM2-MФ, with M1-MФ characterized by increased mRNA- expression and release of IL-6 and TNF-α and M2-MФ by increased IL-10 mRNA expression. Next, we analyzed the expression of VPAC1 in the different BMDM-subtypes. Independent of the mouse genotype (ApoE^−/−^, PACAP^−/−^/ApoE^−/−^), higher VPAC1 mRNA expression was detected in BMDM2-MФ compared with BMDM1-MФ. Interestingly, analysis of VPAC1 protein level showed that PACAP^−/−^/ApoE^−/−^ BMDM had increased VPAC1 protein level compared with ApoE^−/−^. VPAC1 is a member of the GPCR family, whose specific features include the presence of many introns in its gene organization (57, 58). This suggests that alternative splicing events may occur within the VPAC1 receptor, resulting in different VPAC1 splice variants whose functional significance, among others, is not well understood (59). Additionally, Harikumar et al. have shown that VPAC1 can form homodimers (60). In BMDM cells expressing VPAC1, anti-VPAC1 recognized two broad bands with apparent molecular weights of 70 kDa, but not 140 kDa (Additional File Figure S2). Therefore, in this study, only the bands of 70 kDa were evaluated semiquantitative. Previous studies on CHO cell lines showed that bands with molecular weight between 45 and 100 kDa indicate non-glycosylated (ng)-VPAC1 receptor (61). It is also known that the VPAC1 receptor, with the help of its ligand VIP, can be internalized from the plasma membrane via endosomes and transported to the nuclear membrane or cytoplasm, where it is functional or proteasomal degraded (62–66). Thus, the regulation of the VPAC1 receptor is complex and needs to be further investigated concerning the influence of PACAP to understand the differences between mRNA expression and protein analysis.

PACAP, along with VIP, is an important factor in the balance between proinflammatory and anti-inflammatory mediators by inhibiting LPS-induced production of IL-6, IL-12, TNF-α, and NO *in vitro* and *in vivo* and can protect mice from endotoxic shock (67, 68). In our study, PACAP-deficiency in ApoE^−/−^ mice resulted in an increased TNF-α mRNA expression in BMDM1-MΦ and a decreased TNF-α release in BMDM2-MΦ. However, it could not be shown, that PACAP deficiency in ApoE^−/−^ BMDM affect the expression or release of IL-6 or the anti-inflammatory cytokine IL-10. Likewise, this study did not show any effect of oxLDL on the expression of VPAC1 and mRNA expression or release of IL-10, TNF-α, or IL-6 in ApoE^−/−^ or PACAP^−/−^/ApoE^−/−^ BMDM1- and M2-MΦ. It is known, that PACAP controls inflammatory processes by inhibiting NF-κB transcriptional activity in mouse MΦ and LPS-induced THP-1 monocytes (67, 69–71). More precisely, the inhibition of the p65 nuclear translocation and subsequent DNA binding is mediated through the VPAC1 receptor and a non-cAMP transduction pathway (71). Previous publications demonstrate that PACAP inhibits IL-6 production in LPS-induced peritoneal MΦ of mice (72) but, on the other hand, enhances IL-6 release in resting peritoneal MΦ of mice (73). Martinez et al. (73) postulated that the dual effect of PACAP on IL-6 release would be important for immune homeostasis (72, 73). Moreover, previous studies have described the inhibition of LPS-induced IL-6 release by the PACAP-specific receptor PAC1 and that IL-6 stimulation by PACAP is dependent on VPAC1 (72, 73).

PACAP is thought to play a role in lipid metabolism, as previous studies have demonstrated an accumulation of lipids in heart tissue, skeletal muscle, and liver tissue (74). With regard to atherosclerosis, lipid uptake and intracellular accumulation are early events in the development of atherosclerosis, including atherogenic oxLDL causing lipid accumulation that contributes to the formation of foam cells in the intima (75). This modified LDL can be uninhibitedly taken up by MΦ based on scavenger receptors or pinocytosis (75, 76), by which oxLDL uptake was primarily mediated via CD36 (77). In our study, CD36 and LOX-1 mRNA expressions were detected in both BMDM subtypes. Moreover, this study showed that BMDM2-MФ had significantly increased CD36 mRNA expression after oxLDL incubation independent of the genotype of mice. The fact is that endocytosed oxLDL triggers important and complex transcriptional changes in MΦ, including upregulation of CD36 expression (78). Interestingly, in this regard, anti-inflammatory M2-MΦ are more prone to foam cell formation than pro-inflammatory M1-MΦ (79) possibly due to oxLDL-induced upregulation of scavenger receptor CD36 in M2-MΦ. LOX-1 was predominantly expressed in BMDM1-MФ independent of PACAP-deficiency or oxLDL. LOX-1 is a type II integral membrane glycoprotein oxLDL-binding receptor in endothelial cells, which, however, can be up-regulated in MΦ during atherosclerosis (80, 81). Moreover, the lipid accumulation and foam cell formation in the different MΦ-subtypes are diversely discussed, because oxLDL can also be internalized via alternative pathways like micropinocytosis or other scavenger receptors (75, 82). Additionally, Endemann et al. (83) showed that blocking the CD36 functional site with the mouse monoclonal antibody OKM5 in PMA-treated THP-1 resulted only in a 52% reduction of oxLDL-binding (83). In hyperlipidemic CD36^−/−^/ApoE^−/−^ mice the aortic sinus lesions were characterized by electron microscopy and immunohistochemistry and showed an abundance of MΦ foam cells, indicating that lipid uptake by intimal MΦ may occur in the absence of CD36 or SR-A, too (76). In addition, it is entirely unclear what role the intracellular VPAC1 receptor plays in foam cell formation or lipid uptake and accumulation. It is also completely unclear how the relationship between oxLDL, PACAP and the VPAC1 receptor is. A previous study described PACAP as a critical regulator of lipid and/or carbohydrate metabolism (74). Using the detection method of BODIPY™ 493/503, a lower accumulation of neutral lipids was detected in PACAP deficient ApoE^−/−^ BMDM. On the other hand, analyses of fluorescence images (BODIPY™ 493/503) showed that LDs were more concentrated at the cell edge and had lager shape in PACAP^−/−^/ApoE^−/−^ BMDM1-MΦ than in ApoE^−/−^ BMDM1-MΦ, where LDs were located in the periphery of the nucleus and smaller shape. Therefore, PACAP^−/−^/ApoE^−/−^ BMDM1-MΦ showed a more foam cell-like morphology than ApoE^−/−^ BMDM1-MΦ. Considering the data, this possibly indicates an impaired lipid buffer function via LDs and thus an increased risk of lipotoxicity in PACAP^−/−^/ApoE^−/−^ BMDM1-MФ (43). Previous studies showed that PACAP38 reduced lipid accumulation in human THP-1 M0-MФ (29) and that VIP impairs the formation of foam cells by increasing cholesterol efflux in mice MΦ (46). The expression of adipose differentiation-related protein (ADFP) showed that PACAP deficiency in ApoE^−/−^ BMDM increased the ADFP protein level. So far described that the downregulation of ADFP inhibits lipid droplet accumulation and thus reduces the probability of conversion of MФ into foam cells (84, 85). With this in mind, we investigated the VIP/PACAP receptor VPAC1 with regard to intracellular cholesterol and triglyceride concentrations after oxLDL treatment of human THP-1 M1- and M2-MΦ. In our study, intracellular triglyceride concentrations were significantly increased in both THP-1 MΦ subtypes after incubation with oxLDL or oxLDL in combination with the VPAC1 antagonist PG 97–269. Conversely, the VPAC1 agonist [Ala11,22,28]VIP decreased the oxLDL-induced increase in triglyceride concentration in M2-MΦ. Rasbach et al. (29) showed increased plasma triglyceride levels after CED feeding to PACAP^−/−^/ ApoE^−/−^ mice compared with ApoE^−/−^ mice (29). From these data, we conclude that PACAP and especially VPAC1 play important roles in regulating plasmatic and intracellular lipid homeostasis and foam cell formation. Further studies are needed to analyze the interplay of the individual components better.

## 5. Conclusions

The present data provide the background for further research on VPAC1 and PACAP38 as anti-atherogenic therapeutics, because PACAP deficiency impairs luminal stenosis in the aortic arch of ApoE^−/−^ mice as well as VPAC1 receptor expression, inflammatory processes, and lipid homeostasis in M1/M2-MФ.

## Data Availability

The original contributions presented in the study are included in the article/**Supplementary Material**, further inquiries can be directed to the corresponding author.

## Supplementary material

Additional File Sfigure 1. Characterization of human THP-1 M1-/M2-MΦ. PMA-differentiated THP-1 MΦ (M0-MΦ) were incubated with IFN-γ / LPS or IL-4 / IL-13 for 24 h to differentiated to M1- or M2-MΦ. mRNA expressions of (A) CCR7 and (B) CCL17 were determined by qRT-PCR and normalized against GAPDH expression. M1-MΦ was set 100%. (C) TNF-α and (D) IL-10 releases were determined by ELISA. Results are expressed as means + SD. (n = 4). *p ≤ 0.05, ***p ≤ 0.001 vs. M1-MΦ.

Additional File Sfigure 2. Representative western blot of VPAC1 and α-tubulin. BMDM1- and BMDM2-MΦ of ApoE^−/−^ and PACAP^−/−^ / ApoE^−/−^ treated with or without oxLDL.
