# Supplementary figures and images for: PACAP regulates VPAC1 expression, inflammatory processes and lipid homeostasis in M1- and M2-macrophages
Source: Front Cardiovasc Med. 2023 Oct 13;10:1264901. doi: 10.3389/fcvm.2023.1264901 (PMC10611464; doi:10.3389/fcvm.2023.1264901)

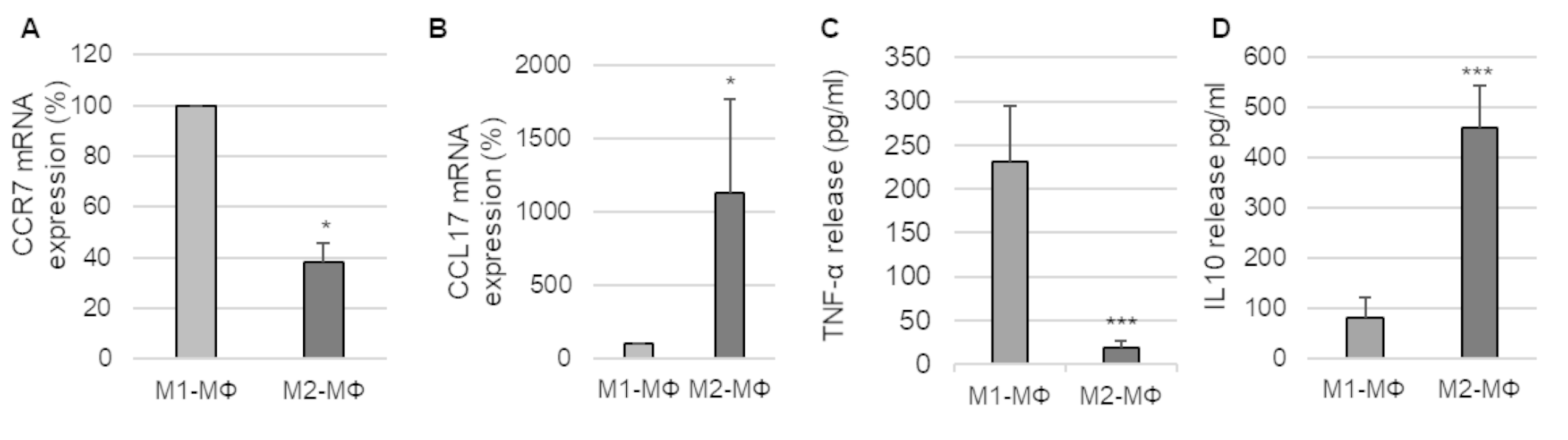

Supplement: Additional File Sfigure 1. — Characterization of human THP-1 M1-/M2-MΦ. PMA-differentiated THP-1 MΦ (M0-MΦ) were incubated with IFN-γ / LPS or IL-4 / IL-13 for 24 h to differentiated to M1- or M2-MΦ. mRNA expressions of (A) CCR7 and (B) CCL17 were determined by qRT-PCR and normalized against GAPDH expression. M1-MΦ was set 100%. (C) TNF-α and (D) IL-10 releases were determined by ELISA. Results are expressed as means + SD. (n = 4). *p ≤ 0.05, ***p ≤ 0.001 vs. M1-MΦ. [file Image1.tiff]

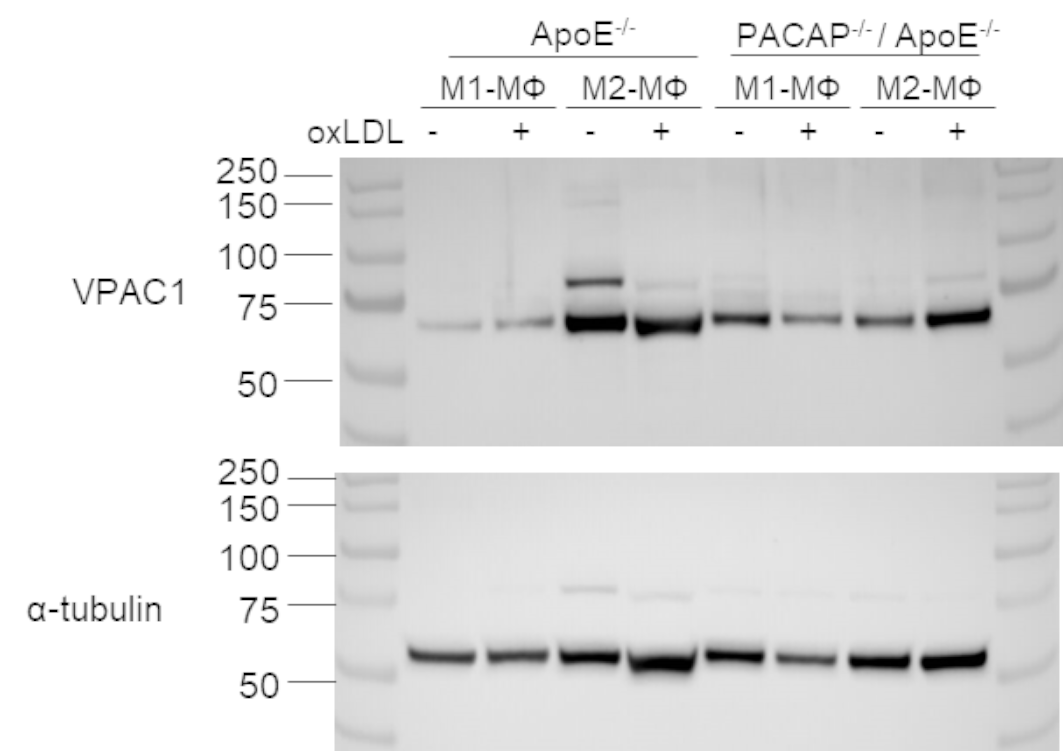

Supplement: Additional File Sfigure 2. — Representative western blot of VPAC1 and α-tubulin. BMDM1- and BMDM2-MΦ of ApoE−/− and PACAP−/− / ApoE−/− treated with or without oxLDL. [file Image2.tiff]
